# Seven gene deletions in seven days: Fast generation of *Escherichia coli* strains tolerant to acetate and osmotic stress

**DOI:** 10.1038/srep17874

**Published:** 2015-12-08

**Authors:** Sheila I. Jensen, Rebecca M. Lennen, Markus J. Herrgård, Alex T. Nielsen

**Affiliations:** 1 The Novo Nordisk Foundation Center for Biosustainability, Technical University of Denmark, Hørsholm, Denmark

## Abstract

Generation of multiple genomic alterations is currently a time consuming process. Here, a method was established that enables highly efficient and simultaneous deletion of multiple genes in *Escherichia coli*. A temperature sensitive plasmid containing arabinose inducible lambda Red recombineering genes and a rhamnose inducible flippase recombinase was constructed to facilitate fast marker-free deletions. To further speed up the procedure, we integrated the arabinose inducible lambda Red recombineering genes and the rhamnose inducible FLP into the genome of *E. coli* K-12 MG1655. This system enables growth at 37 °C, thereby facilitating removal of integrated antibiotic cassettes and deletion of additional genes in the same day. Phosphorothioated primers were demonstrated to enable simultaneous deletions during one round of electroporation. Utilizing these methods, we constructed strains in which four to seven genes were deleted in *E. coli* W and *E. coli* K-12. The growth rate of an *E. coli* K-12 quintuple deletion strain was significantly improved in the presence of high concentrations of acetate and NaCl. In conclusion, we have generated a method that enables efficient and simultaneous deletion of multiple genes in several *E. coli* variants. The method enables deletion of up to seven genes in as little as seven days.

Construction of industrially relevant production strains often requires multiple genomic alterations to direct the carbon flux towards the desired pathways, and to alleviate external stresses accumulating during fermentations. The advent of high throughput sequencing combined with adaptive laboratory evolution^1^, transposon libraries^2^, transcriptomics^3^,^4^,^5^,^6^ and metabolic modelling^7^, has further prompted an increased demand for faster genetic alteration protocols to test and verify combinatorial physiological interactions. In *Escherichia coli*, lambda Red recombineering has been developed to make insertions, deletions and mutations^8^,^9^,^10^,^11^. Similar methods have also found applications in a variety of other bacteria^12^,^13^,^14^. Recently, several methods using improved single stranded oligo recombineering approaches have emerged^8^,^15^,^16^,^17^,^18^. Single stranded oligo recombineering works relatively efficiently for small alterations, however deleting and inserting longer fragments is more efficient when using double stranded recombineering in combination with a selection marker. Furthermore, single stranded oligo recombineering is inefficient when the generated deletions result in a growth disadvantage. Double stranded lambda Red recombineering requires the expression of three lambda-derived proteins. The Gam protein minimizes the degradation of introduced double stranded (ds) DNA recombineering cassettes through interaction with RecBCD and SbcCD nucleases^19^,^20^,^21^. The lambda exonuclease (Exo) degrades the introduced dsDNA in the 5′ to 3′ direction leaving behind a single stranded (ss) DNA^22^,^23^, while the ssDNA binding Beta protein anneals to the Exo generated ssDNA and facilitates its incorporation into genomic DNA during replication^24^,^25^,^26^. The dsDNA recombineering efficiency is affected by multiple factors such as growth phase, transformation efficiency, induction times, homology arms’ length and dsDNA concentration^27^,^28^. Recently, phosphorothioate bonds, which protect dsDNA from exonuclease degradation, have been shown to further increase dsDNA recombineering efficiency, when protecting the lagging strand-targeting dsDNA rather than the leading strand-targeting dsDNA^29^,^30^. This is in analogy to what has been shown for lagging strand preference in ssDNA recombineering^9^.

Removal of the integrated selection marker(s), which is necessary for marker recycling, is often done using FRT-flanked antibiotic cassettes in combination with flippase (FLP) recombinase expression, although alternatives like Cre/loxP and I-SceI have been used as well^31^,^32^. FLP is a bidirectional tyrosine recombinase derived from a 2 μm plasmid present in the nucleus of *Saccharomyces cerevisiae*^33^. FLP recognizes a 34 bp (minimum) target site (FRT), and the binding of two dimers catalyze the cutting and rejoining of inversely repeated FRT sites, which leaves behind a scar containing a single FRT site^34^,^35^,^36^. The most widely used protocol for successive integrations and removal of FRT flanked antibiotic cassettes requires using two temperature sensitive helper plasmids^37^. This results in a time consuming method where repetitive plasmid curing needs to be performed.

In this study we aimed to develop a protocol that enables the creation of multiple deletions in a few days using a combination of dsDNA lambda Red recombineering and flippase recombinase mediated excision of integrated antibiotic markers. The developed methods were used to create multiple combinatorial deletion mutants based on data from recently published transposon library selections^38^. Combinatorial deletion mutants were found to be resistant to high concentrations of acetate, NaCl, and a combination of the two, which is a condition that is often prevalent in high cell density fermentations.

## Results

### Markerless deletions using pSIJ8

**Figure 1:**
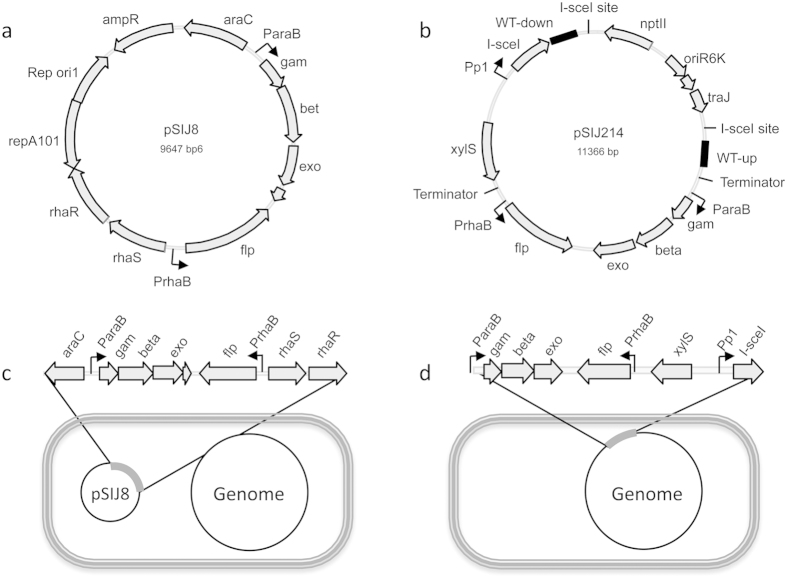
Recombineering systems developed in this study. (**a**) Temperature sensitive plasmid, pSIJ8, with arabinose inducible lambda Red recombineering genes and rhamnose inducible flippase recombinase. (**b**) Integrative plasmid, pSIJ214, containing arabinose inducible lambda Red recombineering genes, rhamnose inducible flippase recombinase and an m-toluic acid inducible homing endonuclease (I-SceI). (**c**) Illustration of cell with pSIJ8, with functional elements highlighted. (**d**) Illustration of the genomically integrated system, with the promoters and genes left in the genome after I-SceI excision of the backbone highlighted.

**Figure 2:**
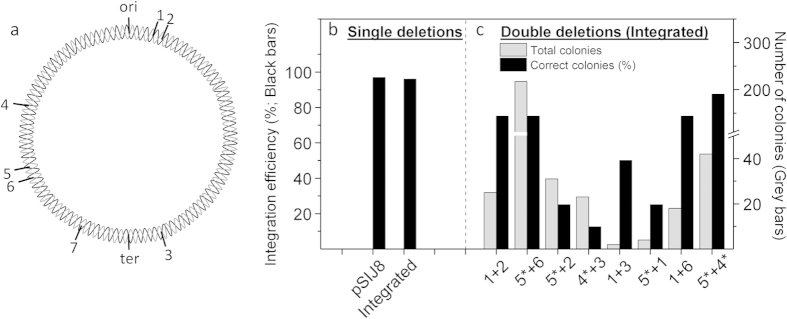
Integration efficiency. (**a**) Genomic location of the different genes targeted for deletion in this study. 1. rfe. 2. typA; 3. yciW; 4. ptsP*; 5. evgA*; *6. ackA*; *7. yobF* (* indicates that the deletion cassette was targeted with both phosphorothioated and phosphorylated primers). (**b**) Efficiency of single deletions using either pSIJ8 or the integrated system in strain SIJ488 (n > 200). (**c**) Efficiency of deletion of two genes during one round of electroporation. Grey bars indicate the total number of colonies obtained; black bars indicate the percentage of correct colonies (n~8, see text).

In order to generate a faster protocol for multiple deletions in *E. coli*, we first constructed the temperature sensitive plasmid *pSIJ8*, which contains the lambda Red recombineering genes (*exo*, *bet* and *gam*) and a flippase (FLP) recombinase. The lambda Red recombineering genes are controlled by an arabinose inducible promoter, which is repressed by AraC in the absence of arabinose. A rhamnose inducible promoter that is positively regulated by RhaRS in the presence of rhamnose controls the flippase recombinase transcription (Fig. 1a,c). The protocol for generation of knockout mutants using PCR products containing 50 bp homology arms is quite similar to the method described by Datsenko and Wanner, 2000^37^. However rather than plating the recovered cells overnight on single antibiotic plates at 37 °C to lose the helper plasmid, the cells were plated on antibiotic plates (km, cm or gm) + LBamp at 30 °C. The following day, single colonies were verified by colony PCR, and >95% of the colonies that were checked using this procedure (>200) contained the correct knockout mutation. A faint band corresponding to the size of the FLP excised knockout cassette was often visible. Although this suggests some leakiness of the rhamnose promoter, it did not appear to affect efficiency. To remove the antibiotic cassettes, single colonies were first grown in e.g. LBkm+amp for two hours in order to minimize the possible occurrence of surviving non-growing satellite cells as well as to reach a cell density appropriate for the subsequent FLP recombineering procedure (see below). This was done at the same time as the colony PCRs. Cell suspensions of positive clones were spun down and cell pellets were re-suspended in LBamp + rhamnose and grown at 30 °C for 4–6 h prior to plating on LBamp plates and growth overnight at 30 °C. The following day, single colonies were checked by colony PCR to verify knockout cassette excision. 96% of the colonies tested (>200) had lost the cassette and were ready for another round of recombineering (Fig. 2b). The procedure took three days to create one markerless mutation and only 16 days to create seven markerless deletions. This included the final plasmid removal, which can be easily done by growing the cells without antibiotics at 37–42 °C. We used this plasmid-based protocol to make further deletions (up to seven in total) in *E. coli* W mutant strains previously generated^38^.

### Markerless deletions using strain SIJ488

The relatively slow growth rate of *E. coli* at 30 °C and the concomitant antibiotic use to maintain the plasmid (pSIJ8) precluded the development of a protocol where the integrated antibiotic cassette could be excised and another antibiotic cassette inserted in one day within reasonable working hours. To circumvent this limitation, we sought to test whether an integrated version of the lambda Red recombineering genes and the flippase recombinase could be used for this purpose. We first made a plasmid that facilitated insertion of the system into the *E. coli* K-12 MG1655 genome (Fig. 1b,d). This plasmid contains arabinose inducible lambda Red recombineering genes, rhamnose inducible flippase recombinase, and an m-toluic acid inducible homing endonuclease, I-SceI. The plasmid pEMG^39^, which contains two endonuclease recognition sites flanking the backbone to be removed, was used as a template for this purpose. The strain carrying the integrated system was used to optimize a protocol for sequential removal of integrated cassette(s) and further deletions in a single day (see below).

### Simultanous knockouts

In order to test whether it is possible to make two simultaneous knockouts during one round of electroporation, we first generated single knockout strains with different antibiotic cassettes. The efficiency of generating and removing single knockouts was similar to that observed for the plasmid-based version (Fig. 2b, 3a). Using genomic DNA from these single knockout strains as templates, we amplified the cassettes with primers resulting in either 50 bp or 100 bp homology arms. To potentially increase the efficiency, we further protected one of the 5′ ends of the dsDNA cassettes using phosphorothioate bonds. This is a strategy that should facilitate preferential generation of lagging-targeting ssDNA cassettes *in vivo*^29^,^30^ since preferential degradation has been shown *in vitro* when using phosphorothioate bonds^40^. We further tested the use of 5′-phosphorylated primers in the opposite end, which should speed up exonuclease degradation^22^. Using cassettes with 50 bp and ~100 bp homology arms without phosphorothioate bonds was not efficient and only generated two double knockout mutants (Δ*rfe*::km + Δ*typA*::cm and Δ*ackA*::km + Δ*evgA*::cm) out of 8 combinations tested. Similar results were obtained using PCR products with 50 bp homology arms and primers containing phosphorothioate bonds in the 5′ end that should preferentially generate lagging strand-targeting ssDNA cassettes *in vivo* (data not shown). In contrast, when using 100 bp homology arms with phosphorothioate bonds in the 5′ end of the PCR product, we were able to make two simultaneous knockouts more or less consistently with 9 out of 11 combinations tested showing positive dual antibiotic resistance. Between 12.5% and 87.5% of the dual antibiotic resistant colonies tested by colony PCR contained the correct knockout mutations (8 colonies per double mutation were tested, except for Δ*rfe*::km + Δ*yciW*::cm (2 colonies) and Δ*evgA*::km + Δ*rfe*::cm (4 colonies)). The results from the tested combinations along with a circle map displaying the genomic location of the different genes is shown in Fig. 2a,c. The combination of asymmetrically phosphorothioated and phosphorylated 5′-ends did show a potential to increase efficiency, however this was not investigated in detail.

### Simultanous removal of dual cassettes

**Figure 3:**
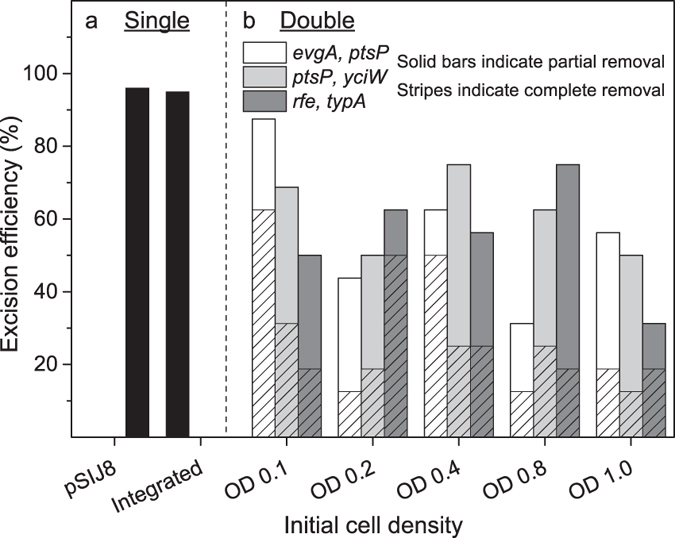
Excision efficiency. Efficiency in percent of (**a**) single excision of integrated antibiotic cassettes using either pSIJ8 or the integrated system in strain SIJ488 (n > 200). (**b**) Simultaneous excision of different integrated antibiotic cassettes after flippase recombinase induction with 50 μM rhamnose for 4 hours in strain SIJ488, using different initial inoculation densities (white *evgA*+*ptsP*; light grey *ptsP*+*yciW*; dark grey: *rfe*+*typA*). Stripes indicate total removal of both cassettes, solids indicate partial removal of both cassettes (n = 16).

The simultaneous removal of dual integrated FRT-flanked antibiotic cassettes using short-term FLP induction (4–6 h) required some optimization. Initial tests had shown that growth at 30 °C was more optimal than at 37 °C for this step (data not shown); therefore 30 °C was used for this procedure. Another preliminary test using different concentrations of rhamnose, variable induction times, and different inoculation densities indicated that the rhamnose concentration did not appear to influence efficiency over a range from 15–75 mM rhamnose, whereas long induction times during growth in LB (16 h) appeared to decrease efficiency somewhat (Supplementary Fig. S1). Interestingly, inoculation densities appeared to influence short-term induction efficiency, so we decided to investigate this further using 50 mM rhamnose and 4 h induction time prior to plating. Three pairs of double mutants were chosen for this purpose (Δ*evgA*::km + Δ*ptsP*::cm; Δ*ptsP*::km + Δ*yciW*::cm and Δ*rfe*::km + Δ*typA*::cm), and some variability was observed between different double mutants. Complete removal of both cassettes (i.e. no visible bands corresponding to the band size expected for the integrated cassettes) was obtained for 13–63% of the cells, whereas partial removal (i.e. a faint but visible band corresponding to the expected band size of either one of the integrated cassettes) was obtained for 44–88% of the cells using an initial inoculation OD of 0.1–0.4. Lower overall efficiency was generally observed for inoculation ODs >0.4 (Fig. 3b).

### Successive removal and integration of cassette(s) in one day

The high efficiency of integration and removal of one antibiotic cassette (>95%) and the somewhat lower but still relatively efficient integration and removal of dual antibiotic cassettes during short term induction, allowed for the opportunity to develop a method where integrated cassette(s) could be removed and new cassette(s) could be integrated within the same day. This was possible using a 2:1:2 protocol whereby either two cassettes were removed and one more deletion were made in one day, or one cassette was removed and two more deletions were made in one day. This method was highly efficient for some combinations tested (e.g. Δ*rfe*::km + Δ*typA*::cm and Δ*ackA*::km + Δ*evgA*::cm), which facilitated the generation of seven deletions in seven days. However, in some cases no clones could be found with either integrated or removed dual cassettes. A more robust method for most combinatorial deletions is thus to simply remove one cassette and integrate only one additional cassette in the same day in a 1:1 procedure. Using the 1:1 approach, seven deletions take nine days to make rather than seven. We used the 2:1:2 protocol to generate multiple deletion mutants (up to seven in total) in *E. coli* K-12 MG1655 with the aim of identifying strains with improved tolerance towards stresses typically encountered during fed batch fermentation.

### Phenotypic characterization of multiple deletion strains

Several gene deletions involved in tolerance towards acetate, osmotic stress, combined acetate and osmotic stress, and combined low pH and osmotic stress were originally discovered in transposon library selections of *E. coli* W^38^. In that study all double knockout combinations of single beneficial deletions were tested and those with negative epistatic interactions were eliminated prior to triple knockout constructions. However due to the time involved in constructing knockouts with traditional lambda Red recombineering using pSIM5^41^, several combinatorial deletions were not tested. Additionally, in experiments where short-term selection of a Tn5 library in K-12 MG1655 Δ*hsdR* in M9 and M9 containing high acetate, high salt, and high acetate and salt concentrations were followed by next-generation sequencing of Tn5-chromosome junctions, it was found that several of these gene knockouts also improve K-12 MG1655 fitness under those conditions (unpublished data). These included loss-of-function of *ptsP, yobF*, and *ackA* for improved growth in high acetate concentrations, and loss-of-function of *ackA* for improved growth in high acetate and high salt concentrations. To further explore combinations of mutations in both *E. coli* W and *E. coli* K-12 MG1655, the developed rapid multiple knockout generation methods were used to successfully create several strains that carried between four to seven gene deletions that also avoided most known negative epistatic interactions between gene knockout pairs.

An initial screening of the growth behaviour of different single and triple to septuple deletion strains exposed to 15 g L^−1^ NaAc, 0.6 M NaCl and 5 g L^−1^ NaAc + 0.4 M NaCl was performed using a high throughput optical image scanner (data not shown). The optical image scanning results for cells exposed to high acetate provided relatively smooth growth curves, although some noise was observed for cell cultures exposed to high salt and high salt + acetate. This was likely due to cell/salt aggregate formation, which prohibited accurate growth parameter extraction. Nevertheless, based on the optical image scanning results we were able to select the best single and combinatorial deletion strains for more carefully controlled experiments using the Biolector microbioreactor system.

**Figure 4:**
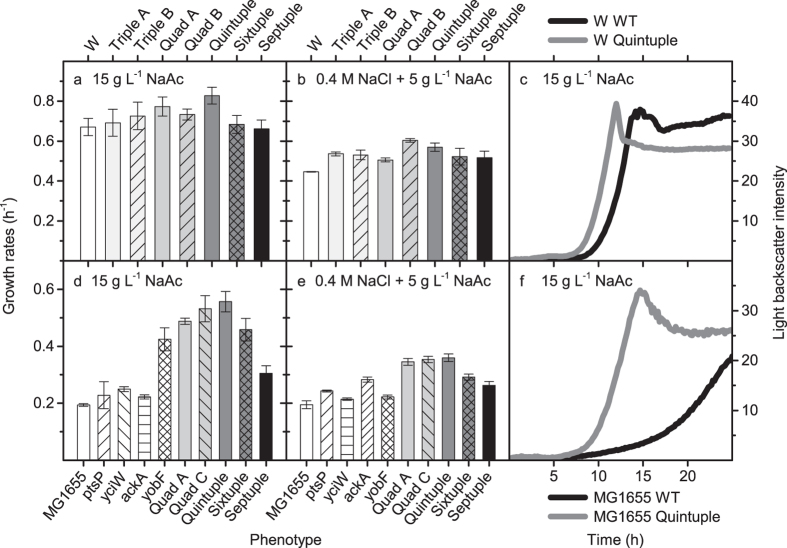
Growth rates and growth curves of *E. coli* W (a–c) and *E. coli* K-12 MG1655 (d–f) and derivatives containing different deletions, when exposed to 15 g L^−1^ NaAc (a,c,d,f), or 0.4 M NaCl + 5 g L^−1^ NaAc (b,e). Triple A (*evgA*; *ptsP*; *yciW*) Triple B (*evgA*; *ptsP*; *yobF*), Quad A (*evgA*; *ptsP*; *yciW*; *ackA*), Quad B (*evgA*; *ptsP*; *yciW*; *yobF*), Quad C (*evgA*; *ptsP*; *yobF*; *ackA*), Quintuple (*evgA*; *ptsP*; *yciW*; *ackA*; *yobF*), Sixtuple (*evgA*; *ptsP*; *yciW*; *typA*; *yobF*; *ackA*), Septuple (*evgA*; *ptsP*; *yciW*; *ackA*; *typA*; *yobF*; *rfe*).

From this screening, the highest growth rate for cells exposed to high acetate was obtained for a quintuple deletion strain (Δ*evgA*; Δ*ptsP*; Δ*yciW*; Δ*ackA*; Δ*yobF*) in both *E. coli* W and *E. coli* K-12 MG1655. Several other single and combinatorial deletion mutants also exhibited significantly (p < 0.05) higher growth rates compared to wild type strains when exposed to this condition. Furthermore, the *E. coli* W quintuple deletion strain displayed significantly higher growth rates when compared to the triple deletion strains tested, and the *E. coli* K-12 MG1655 quintuple deletion strain had significantly higher growth rates when compared to the best single deletion strain (Δ*yobF*::km) investigated (Fig. 4a,d). For cells exposed to high salt conditions (0.6 M NaCl), an *E. coli* K-12 MG1655 single deletion strain (*ptsP*::km) displayed significantly higher growth rates as compared to the parental strain. The growth rates of the quadruple and quintuple deletion strains tested were similar to wild type in both strain backgrounds, whereas the growth rates of *E. coli* K-12 MG1655 hextuple (Δ*evgA*; Δ*ptsP*; Δ*yciW*; Δ*ackA*; Δ*typA*; Δ*yobF*) and septuple (Δ*rfe*; Δ*typA*; Δ*yciW*, Δ*ptsP*, Δ*evgA*, Δ*ackA*, Δ*yobF*) deletion strains were significantly lower (Fig. 4b,e). Most deletion strains showed significantly higher growth rates when exposed to the combinatorial stress of high acetate and high salt concentrations. The highest growth rate in the different strain backgrounds was observed for an *E. coli* W quadruple (Δ*evgA*; Δ*ptsP*; Δ*yciW;* Δ*yobF*) deletion strain and an *E. coli* K-12 MG1655 quintuple (Δ*evgA*; Δ*ptsP*; Δ*yciW*; Δ*ackA*; Δ*yobF*) deletion strain (Fig. 4c,f). Overall, the combinatorial deletions were found to provide a significant improvement in growth rates of *E. coli* K-12 MG1655 in all the conditions tested except for high salt concentration. A quadruple knockout additionally showed a nearly 3-fold increase in growth rate (from 0.17 h^−1^ to 0.50 h^−1^) in pH 5.5 + 0.4 M NaCl, which was higher than any single knockout strain (P < 0.10) (data not shown).

## Discussion

Construction of industrially relevant production strains often requires multiple genomic alterations to increase the flux towards precursors and/or products, and to alleviate e.g. product toxicity. In *E. coli* the most widely used protocol for rapid generation of genomic alterations is lambda Red recombineering. Recently, several efforts have been made to improve the efficiency of single-stranded oligo recombineering^8^,^15^,^16^,^17^,^18^, which is an approach that is difficult to use when deleting larger fragments, and/or when deleting functions that result in a growth retarded phenotype.

In this study, we focused on improving existing technologies for the combined action of lambda Red recombineering with dsDNA and flippase mediated removal of resistant markers, in order to enable fast generation of multiple deletion strains. We first developed a protocol based on an easily curable temperature sensitive plasmid containing the lambda Red recombineering genes and a flippase recombinase. This enabled continuous integration and removal of cassettes without the concomitant need of re-transforming plasmids. The developed protocol results in a much faster procedure for generating multiple deletions as compared to the rather cumbersome, but commonly used, two-plasmid based protocol^37^. A similar method has recently been described that uses an analogous plasmid containing the lambda Red recombineering genes and a Cre-recombinase^31^. Similarly, an analogous plasmid used for performing recombinations on plasmids contains rhamnose inducible lambda Red recombineering genes and a tetracycline inducible debilitated flippase recombinase^42^. Since our method uses the flippase recombinase FLP, it can, in contrast to the plasmid based on Cre-recombinase, be used in combination with the Keio collection of single gene deletion mutants^43^,^44^. We furthermore developed a protocol which facilitated very short flippase recombinase induction times (4 hours) to remove the integrated antibiotic cassettes in contrast to the overnight induction previously described using a similar plasmid^42^. This short induction time can be important when generating multiple deletion strains as it decreases the possibility of recombineering events and inversions caused by the presence of old scars. The short induction time needed in our protocol prompted us to develop a method where integrated cassettes could be removed and a new deletion could be done in the same day. Because of the relatively slow growth rate of *E. coli* at 30 °C as well as the fitness costs related to plasmid maintenance and antibiotic exposure, we first integrated the recombineering genes into the *E. coli* K-12 MG1655 genome. The integrated recombineering genes worked just as efficiently as the plasmid based version with regards to integration and removal of antibiotic cassettes, and it facilitated a protocol where integrated cassette(s) could be removed and new gene(s) could be deleted in the same day. Using the integrated version in combination with the protocol developed that facilitated short time induction of the flippase recombinase, we were thus able to make at least twice as many deletions within the same amount of time as the plasmid based versions described above^42^. The integrated version further facilitates pre-screening of strains without having to remove the recombineering plasmid first, as long as arabinose or rhamnose is not used as inducers for gene expression.

To further speed up the procedure, we tested whether it would be possible to make two deletions during one round of electroporation using dual antibiotic markers in combination with PT-bonds in one end of the cassettes to be integrated. PT-bonds have been shown to specifically inhibit the action of exo-nuclease degradation *in vitro*^40^. Phosphorothioate bonds in the 5′ end have been shown to improve double stranded lambda Red recombineering efficiency, likely because they facilitate preferential generation of lagging-targeting ssDNA-cassettes^29^,^30^. Our study supports this observation, as we were unable to consistently create double mutants using primers without phosphorothioate bonds except for genes located in close proximity to each other. The latter observation is consistent with the fact that multiplex single-stranded oligo recombineering efficiency has been shown to be increased if the targets are in relatively close proximity to each other^45^. Furthermore our results suggest that degradation of the dsDNA cassettes or the *in vivo* generated ssDNA strands might be a limiting factor for the overall efficiency in combination with a permissive replication fork. It is therefore beneficial to carefully study the genomic positions of genes to be altered before starting to make strains that require multiple genomic alterations. When testing simultaneous removal of integrated antibiotic cassettes, we found that long induction times (16 h) were less efficient than shorter induction times. We speculate that this may be due to possible recombineering events between distantly located FRT sites, which could result in a growth advantage for cells that did not induce the recombinase gene. We furthermore found that relatively dense inoculation densities improved the efficiency.

We used the developed methods to successfully create several strains carrying from four to seven gene deletions that could potentially be involved in tolerance towards acetate and high salt concentrations in *E. coli* K-12 MG1655 and *E. coli* W. Deletion of *yobF* showed the highest improvement in tolerance to acetate in *E. coli* K-12 MG1655 of the single deletion strains investigated, whereas deletion of *ptsP* showed the highest improvement in tolerance to high salt conditions. This is to some extent in contrast to what has previously been observed for *E. coli* W, where no significant increases in growth rate were observed for these mutations^38^. However this may simply reflect that the growth rate of wild-type *E. coli* W is much higher overall, including when exposed to these conditions. Positive epistatic interactions were observed in some of the quadruple and quintuple deletion strains investigated. The quintuple deletion strains (*evgA*, *ptsP*, *yciW*, *yobF*, and *ackA*) showed the highest growth rate of all strains tested when exposed to high acetate concentration, whereas further deletion(s) of *typA* and *rfe* exhibited negative epistatic interactions. The combinatorial deletions include: one regulatory protein EvgS (the sensor kinase of the EvgSA two component signal transduction); PtsP, which may play a signalling role; the acetate kinase AckA, which catalyzes the reversible conversion of acetyl phosphate and acetate; as well as two proteins of unknown function (YciW, a predicted oxidoreductase and YobF, a small protein with no known function). It is currently not clear what is causing the observed epistatic interactions. The quintuple *E. coli* W deletion strain additionally had significantly higher growth rates when compared to triple deletion strains previously generated^38^. This highlights the need for improved protocols for fast generation of multiple genomic deletion procedures to enable comprehensive screening and assessment of the phenotypic space that subsequently allows for significant improvements of industrially relevant production strains.

## Conclusion and Perspectives

**Figure 5:**
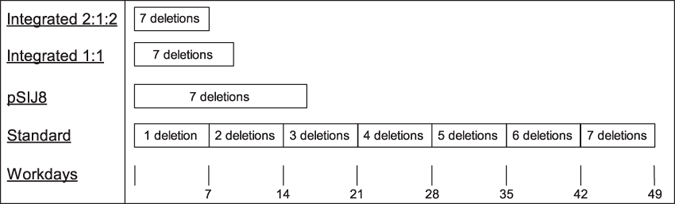
Timeline for generation of deletion mutants. Estimated time for making up to seven markerless deletions using either the integrated 2:1:2 or 1:1 system in strain SIJ488, the one-plasmid procedure using pSIJ8 or the standard two plasmid based procedure.

In this study a protocol was developed that enables the construction of seven deletions in as little as seven days (Fig. 5) based on the combined action of lambda Red recombineering and flippase recombinase mediated excision of integrated antibiotic cassettes. The developed protocol can be used in combination with the Keio collection of single gene deletion strains and can be easily combined with a similar system based on Cre-recombinase mediated excision of antibiotic cassettes. With proper consideration of the genomic locations of desirable modifications, it may furthermore be possible to combine the dsDNA recombineering used in this study with ssDNA recombineering, which could facilitate even faster engineering of multiple genomic alterations.

## Material and Methods

### Strains, media and plasmids

*E. coli* K12 MG1655 and *E. coli* W were grown in LB broth or on LB agar plates supplemented, when needed, with appropriate antibiotics (ampicillin 100 μg mL^−1^ (ap), kanamycin 25–50 μg mL^−1^ (km), chloramphenicol, 20–30 μg mL^−1^ (cm), and gentamycin, 10 μg mL^−1^ (gm)). Cells were grown at either 30 °C or 37 °C. 100 μL liquid LB-amp was spread on km, gm, or cm plates for plasmid maintenance in knockout stains when using pSIJ8. Strains and plasmids used in the developed protocols are listed in Table 1, strains generated for physiological comparisons are listed in Table 2, and precursor strains, primers, and plasmids^37^,^38^,^39^,^46^,^47^,^48^ used as PCR-templates are listed in Supplementary Table S1 and S2.

### Construction of vectors

PCR-templates were generated using pfuX7^49^ and standard PCR conditions. PCR products were in general purified from 1% agarose gels using the Macherey-Nagel Nucloespin gel and PCR clean up kit. USER-cloning was performed by preparing a 12 μL reaction mixture containing 1 μL backbone-PCR-template (10–20 ng μL^−1^), 2–4 μL insert(s) (size/concentration-dependent), 1.2 μL T4-ligase buffer, 1 μL USER enzyme, and DNAse/RNAse free water. The mixture was incubated for 25 min at 37 °C and 25 min at 25 °C, after which 8 μL DNAse/RNAse free water was added. 5 μL of this mixture was used to transform 50 μL chemically competent *E. coli* DH5α-λPIR cells or chemically competent XL-1 blue cells following standard procedures. Plasmids were purified using the Macherey-Nagel Nucleospin plasmid DNA purification kit, and then sequenced.

### Genomic integration and removal of integrated deletion system

Arabinose inducible lambda Red recombineering genes (*exo*, *bet*, and *gam*), rhamnose inducible flippase (FLP) recombinase, and m-toluic acid inducible homing endonuclease (I-SceI) were integrated into the *E. coli* MG1655 genome by homologous recombination downstream of the *glmS* gene using pSIJ214. Correct insertion was verified by colony PCR and the integrated backbone was removed by inducing the integrated I-SceI gene with 15 mM m-toluic acid (final concentration) for 16 h at 30 °C, after which the induced culture was plated on LB agar plates and grown overnight at 37 °C. Colonies were re-streaked on LB-km and LB agar plates to verify removal of the backbone. Colony PCRs were performed on positive colonies to check whether the cells had reverted to wild-type or still contained the integrated genes.

### Generation of knockouts

Oligonucleotides used to amplify FRT-flanked selection cassettes are listed in Table S2. Oligonucleotides with or without phosphorothioate (PT) bonds were designed to target single knockout cassettes already integrated in the genome either ~50 bp or 100 bp up- and down-stream of the target genes as described later. Deletions using lambda Red recombineering were performed similarly to previously described methods^37^. Briefly, cells were grown in shake flasks in LBamp at 30 °C or LB at 37 °C to an OD of approximately 0.3. The lambda Red recombineering genes were induced for 30–45 min by adding 15 mM L-arabinose (final concentration). Induced cultures were made electrocompetent using standard procedures, and aliquots of 50 μL electrocompetent cells were mixed with 5 μL PCR-template (~250 ng DNA) and electroporated (1.8 kV, 0.1 cm gap). After electroporation, cells were recovered in 1 mL LB at 30 °C or 37 °C for two hours. Simultaneous deletion of two genes during one electroporation round was made by the same protocol, but using 50–100 bp flanking primers with or without PT-bonds as listed in Table S2, and by mixing the different PCR products prior to electroporation. Removal of antibiotic markers was done at 30 °C in LB by inducing FLP with 50 mM L-rhamnose (final concentration) at an OD~0.1–0.4 for 4–6 h prior to plating unless otherwise stated. Integration of cassettes and removal of resistant markers was verified by colony PCR using OneTaq 2× Master Mix (New England Biolabs, Ipswich, MA), according to manufactures instructions. pSIJ8 were cured from the cells by growing them at 37 °C.

### Pre-screening of growth phenotypes

Seven genes encoding the following proteins: Rfe (involved in enterobacterial common antigen (ECA) and O-antigen LPS biosynthesis), PtsP (part of the nitrogen phosphotransferase system, PTS^Ntr^), YobF (a small protein with no known function), EvgS (the sensor kinase of the EvgSA two component signal transduction system), YciW (a predicted oxidoreductase), AckA (acetate kinase, catalyzing reversible conversion of acetyl phosphate and acetate) and TypA (a member of the ribosome-binding GTPase family), were selected for combinatorial gene deletions (4–7 deletions) based on results from a previous study^38^. The strains carrying multiple deletions used for investigations of various growth parameters are listed in Table 2. Pre-cultures of each strain were inoculated from single colonies on LB agar plates in biological triplicates into 0.3 mL of M9 medium (12.8 g L^−1^ Na_2_HPO_4_ ·7H_2_O, 3.0 g L^−1^ KH_2_PO_4_, 0.5 g L^−1^ NaCl, 1.0 g L^−1^ NH_4_Cl, 2 mM MgSO_4_, 0.1 mM CaCl_2_) supplemented with 0.4% (w/v) glucose, 2 μM thiamine HCl, 60 μM FeCl_3_, and a trace element solution (1.8 mg L^−1^ ZnSO_4_·7H_2_O, 1.2 mg L^−1^ CuCl_2_·2H_2_O, 1.2 mg L^−1^ MnSO_4_·H_2_O, and 1.8 mg L^−1^ CoCl_2_·6H_2_O) in 96-well deep well plates and grown to saturation overnight (~16 to 20 h) in a New Brunswick Innova® 44 plate shaker (Eppendorf, Hamburg, Germany) at 37 °C and 300 rpm. Each culture was diluted 10-fold in supplemented M9 medium, and 30 μL was inoculated into 270 μL of M9 medium containing different stressors (0.6 M NaCl; 15 g L^−1^ NaAc; 0.4 M NaCl and 5 g L^−1^ NaAc) in 96-well square half-deep well plates with transparent glass bottoms (Enzyscreen B.V., Haarlem, The Netherlands). Optical image scanning using a Growth Profiler 1152 (EnzyScreen B.V.) was used to monitor growth. Integrated green pixel values (G-values) from each well were converted to equivalent OD_600_ values using calibration values that were fit to a Monod function (G-value = *a*·OD_600_*/(b+*OD_600_)) with *a* and *b* parameters determined by non-linear regression.

### Screening of growth phenotypes

Pre-cultures of each strain were inoculated in biological triplicates in M9 medium supplemented as described for qualitative growth screening. Following overnight growth to saturation, each culture was diluted 10-fold in deionized water to a final volume of 200 μL in a clear polystyrene 96 well plate, and the OD_600_ was measured using a Synergy Mx plate reader (BioTek Instruments, Winooski, VT). Based on this path length, cells were inoculated to an initial OD_600_ of 0.03 into wells of a 48-well FlowerPlate without optodes (m2p-labs GmbH, Baesweiler, Germany) containing supplemented M9 medium. The final culture volume in each well was 1.4 mL, and the final stressor concentration (when present), was the same as previously described for qualitative growth screening. Cultures were incubated in a BioLector microbioreactor system (m2p-labs GmbH) at 37 °C with 1000 rpm shaking and the light backscatter intensity was monitored.

## Additional Information

**How to cite this article**: Jensen, S. I. *et al.* Seven gene deletions in seven days: Fast generation of *Escherichia coli* strains tolerant to acetate and osmotic stress. *Sci. Rep.* **5**, 17874; doi: 10.1038/srep17874 (2015).

## Supplementary Material

Supplementary Information

## Figures and Tables

**Table 1:** Strains and plasmids for alterations.

Strain	Genotype	Reference
Strains		
* Escherichia coli* K-12 MG1655	F- λ- *ilvG- rfb-50 rph-1*	A. Feist^1^
* Escherichia coli* W	F-, soil isolate, harbors pRK1 and pRK2	DSMZ 1116
SIJ488	*E. coli* K-12 MG1655Tn7::para-exo-beta-gam; prha-FLP; xylSpm-IsceI	This study
**Plasmid**	**Description**	**Reference**
Plasmids for deletions		
pSIJ8	pkd46, rhaRS-prha-FLP, amp	This study
pSIJ214	pEMG, MG1655-tn7-para-exo-beta-gam; prha-flp; xylSpm-I-SceI, km	This study
Plasmids for frt flanked deletion cassettes		
pkd3	Ap^R^, FRT-cm^R^-FRT, *ori*R6K	37
pkd4	Ap^R^, FRT-Km^R^-FRT, *ori*R6K	37
pSIJ196	pEMG amp, frt::spec::frt	This study
pSIJ197	pEMG amp, frt::gm::frt	This study

^1^This strain was generously donated by Dr. Adam Feist, University of California, San Diego.

**Table 2:** Strains generated for physiological comparisons.

Strain		Genotype	Reference
1 deletion strains			
SIJ1057	*E. coli* MG1655	Δ*rfe*::km	This study
SIJ1058	*E. coli* MG1655	Δ*typA*::km	This study
SIJ1059	*E. coli* MG1655	Δ*yciW*::km	This study
SIJ1060	*E. coli* MG1655	Δ*ptsP:*:km	This study
SIJ1061	*E. coli* MG1655	Δ*evgA*::km	This study
SIJ1062	*E. coli* MG1655	Δ*yobF*::km	This study
SIJ1063	*E. coli* MG1655	Δ*ackA*::km	This study
SIJ1071	*E. coli* W	Δ*rfe*::km	This study
SIJ1072	*E. coli* W	Δ*typA*::km	This study
SIJ1073	*E. coli* W	Δ*yciW*::km	This study
SIJ1074	*E. coli* W	Δ*ptsP*::km	This study
SIJ1075	*E. coli* W	Δ*evgA*::km	This study
SIJ1076	*E. coli* W	Δ*yobF*::km	This study
SIJ1077	*E. coli* W	Δ*ackA*::km	This study
3 deletion strains			
RL	*E. coli* W	Δ*evgA*; Δ*ptsP* : Δ*yciW::km*	38
RL	*E. coli* W	Δ*evgA*; Δ*ptsP* : Δ*yobF::km*	38
4 deletion strains			
SIJ1099	*E. coli* W	Δ*evgA*; Δ*ptsP* : Δ*yciW*; Δ*ackA*::km	This study
SIJ1100	*E. coli* W	Δ*evgA*; Δ*ptsP* : Δ*yciW*; Δ*typA*::km	This study
SIJ1101	*E. coli* W	Δ*evgA*; Δ*ptsP* : Δ*yobF*; Δ*ackA*::km	This study
SIJ1102	*E. coli* W	Δ*evgA*; Δ*ptsP* : Δ*yciW*; Δ*yobF*::km	This study
SIJ1115	*E. coli* W	Δ*evgA*; Δ*ptsP*; Δ*yobF*; Δ*typA*::km	This study
SIJ1104	SIJ488	Δ*evgA*; Δ*ptsP*; Δ*yciW*; Δ*typA*	This study
SIJ1105	SIJ488	Δ*evgA*; Δ*ptsP*; Δ*yobF*; Δ*ackA*	This study
SIJ1106	SIJ488	Δ*evgA*; Δ*ptsP*; Δ*yciW*; Δ*ackA*	This study
SIJ1107	SIJ488	Δ*evgA*; Δ*ptsP*; Δ*yciW*; Δ*yobF*	This study
SIJ1113	SIJ488	Δ*evgA*; Δ*ptsP*; Δ*yobF*; Δ*typA*	This study
5 deletion strains			
SIJ1096	*E. coli* W	Δ*evgA*; Δ*ptsP* : Δ*yciW*; Δ*ackA*; Δ*yobF*::km	This study
SIJ1097	*E. coli* W	Δ*evgA*; Δ*ptsP* : Δ*yciW*; Δ*typA*; Δ*yobF*::km	This study
SIJ1098	*E. coli* W	Δ*evgA*; Δ*ptsP* : Δ*yciW*; Δ*ackA*; Δ*typA*::km	This study
SIJ1108	SIJ488	Δ*evgA*; Δ*ptsP* : Δ*yciW*; Δ*ackA*; Δ*yobF*::km	This study
SIJ1109	SIJ488	Δ*evgA*; Δ*ptsP* : Δ*yciW*; Δ*typA*; Δ*yobF:*:km	This study
SIJ1110	SIJ488	Δ*evgA*; Δ*ptsP* : Δ*yciW*; Δ*ackA*; Δ*typA*	This study
6 deletion strains			
SIJ1095	*E. coli* W	Δ*evgA*; Δ*ptsP* : Δ*yciW*; Δ*ackA*; Δ*typA*; Δ*yobF*	This study
SIJ1111	SIJ488	Δ*evgA*; Δ*ptsP* : Δ*yciW*; Δ*ackA*; Δ*typA*; Δ*yobF*	This study
7 deletion strains			
SIJ1103	*E. coli* W	Δ*evgA*; Δ*ptsP*: Δ*yciW*; Δ*ackA*; Δ*typA*; Δ*yobF*; Δ*rfe*::km	This study
SIJ1112	SIJ488	Δ*rfe*; Δ*typA*; Δ*yciW*; Δ*ptsP*; Δ*evgA*; Δ*ackA*; Δ*yobF*::km	This study
