# Supplementary material for: Seven gene deletions in seven days: Fast generation of Escherichia coli strains tolerant to acetate and osmotic stress
Source: Sci Rep. 2015 Dec 8;5:17874. doi: 10.1038/srep17874 (PMC4672327; doi:10.1038/srep17874)
Supplement: Supplementary Information [file srep17874-s1.pdf]

# Seven gene deletions in seven days:
# Fast generation of *Escherichia coli* strains tolerant to acetate and osmotic stress

Sheila I. Jensen[1*], Rebecca M. Lennen[1], Markus J. Herrgård[1], Alex T. Nielsen[1*]

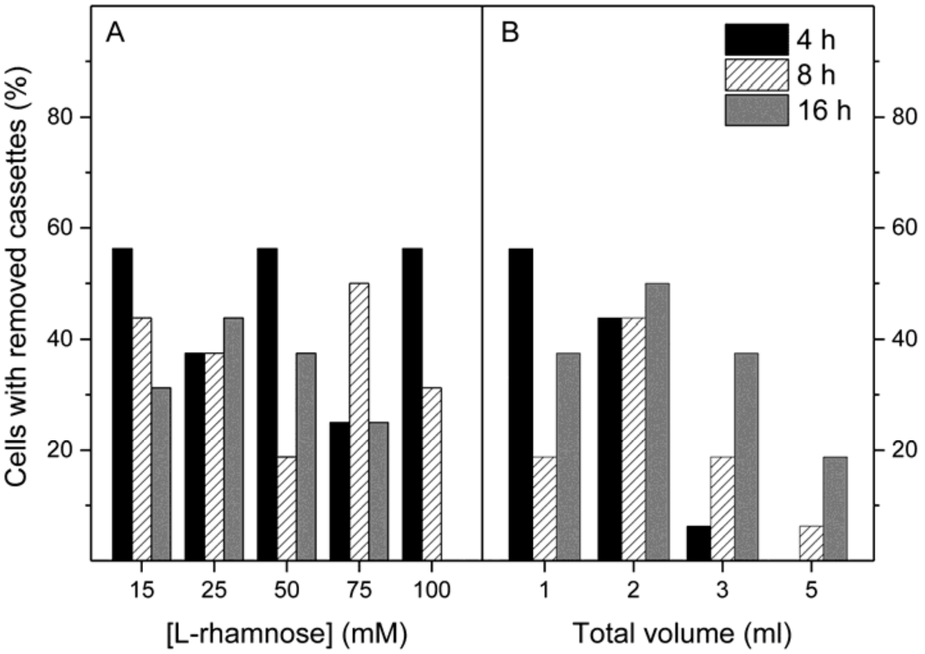


**Supplementary Figure S1.** Efficiency in percent of simultaneous excision of different integrated antibiotic cassettes in strain SIJ488 induced for 4 h (black bars), 8 h (stripes) or 16 h (grey). A) Efficiency using different concentrations of rhammose. B) 50 µl growing cell culture inoculated into 1 ml, 2 ml, 3 ml or 5 ml LB and induced with 50 mM rhamnose (n=16).

**Supplementary Table S1.** Precursor strains and plasmids used in this study.

| Strain | Genotype | Reference |
|---|---|---|
| XL-1 blue | recA1 endA1 gyrA96 thi-1 hsdR17 supE44 relA1 lac [F'proAB lacIqZΔM15 Tn10 (Tetr)] | Agilent technologies |
| DH5α λpir | λ*pir* lysogen of DH5α | V. de Lorenzo[2] |

| Plasmid | Description | Reference |
|---|---|---|
| **Plasmids** | | |
| pKD46 | $Ap^R$, *ori*R101, repA101$^{TS}$, *araC*-ParaB::*gam-beta-exo* | (Datsenko and Wanner 2000) |
| pEMG | $Km^R$, *ori*R6K, *lacZα*, two I-SceI sites | (Martinez-Garcia and de Lorenzo 2011) |
| pSEVA 631 | $Gm^R$, *ori*pBBR1, *oriT* | (Silva-Rocha et al. 2013) |
| pSEVA 441 | $sm^R$, *ori*pRO1600/ColE1, *oriT* | (Silva-Rocha et al. 2013) |
| pFLP2 | $Ap^R$, *ori*1600, *ori*T, rep, cI857-*FLP*, *sacB* | (Hoang et al. 1998) |
| pSW-1 | $Ap^R$, *ori*RK2, *xylS*, Pm-*I-sce*I | (Wong and Mekalanos 2000) |
| pSIJ_180 | pEMG $ap^R$, frt::cm::frt | This study |

[2]This strain was generously donated by V. de Lorenzo, Centro Nacional de Biotecnología-CSIC, Madrid, Spain

Datsenko KA, Wanner BL. 2000. One-step inactivation of chromosomal genes in *Escherichia coli* K-12 using PCR products. Proc. Natl. Acad. Sci. 97(12):6640-6645.

Hoang TT, Karkhoff-Schweizer RR, Kutchma AJ, Schweizer HP. 1998. A broad-host-range Flp-FRT recombination system for site-specific excision of chromosomally-located DNA sequences: application for isolation of unmarked Pseudomonas aeruginosa mutants. Gene 212(1):77-86.

Martinez-Garcia E, de Lorenzo V. 2011. Engineering multiple genomic deletions in Gram-negative bacteria: analysis of the multi-resistant antibiotic profile of *Pseudomonas putida* KT2440. Environ Microbiol 13(10):2702-16.

Silva-Rocha R, Martinez-Garcia E, Calles B, Chavarria M, Arce-Rodriguez A, de Las Heras A, Paez-Espino AD, Durante-Rodriguez G, Kim J, Nikel PI and others. 2013. The Standard European Vector Architecture (SEVA): a coherent platform for the analysis and deployment of complex prokaryotic phenotypes. Nucleic Acids Res 41(Database issue):D666-75.

Wong SM, Mekalanos JJ. 2000. Genetic footprinting with mariner-based transposition in *Pseudomonas aeruginosa*. Proc Natl Acad Sci U S A 97(18):10191-6.

**Supplementary Table S2:** Primers used in this study

| Primers for deletions and verification | Sequence |
|---|---|
| 662_rfe_EC-W-MG_fw | CTTCGTGGTTATACTTCTGCTAATAATTTTCTCTGAGAGCATGCATTGTGTGTAGGCTGGAGCTGCTTC |
| 974_rfe_BL21_fw | CTTCGTGGTTATACTTCTGCCAATAATTTTCTCTGAGAGCATGCATTGTGTGTAGGCTGGAGCTGCTTC |
| 663_rfe_rv | AGGCATTGGTTGTGTCATCACATCCTCATTTATTTGGTTAAATTGGGGCTGCTTGCATATGAATATCCTCCTTAG |
| 664_evgA_fw | ATTACTACAGGGAGAAGGGAGATGCTTCATTGCAAAGGGAATAATCTATGTGTAGGCTGGAGCTGCTTC |
| 665_evgA_W_rv | GAAAAAAATAGGGTAAAAACTTCATGTGATTAGCCGATTTTGTTACGTTGGCTTGCATATGAATATCCTCCTTAG |
| 874_evgA_MG-BL_rv | GAAAAATATAGGGTAAAAACTTCATGTGGTTAGCCGATTTTGTTACGTTGCTTGCATATGAATATCCTCCTTAG |
| 666_typA_fw | TAATCCGTGTACAATAACGCGCTATTTCTAATGCCTGAGGCAAAGTTGTGTGTAGGCTGGAGCTGCTTC |
| 667_typA_rv | GGCAGGTTTTTTATGACTAAAAAACGAAATTAATCGTCTTTCGGTGCGCGGCTTGCATATGAATATCCTCCTTAG |
| 668_yobF_fw | TTGTATGTGATAACAGATTTCGGGTTAAACGAGGTACAGTTCTGTTTATGTGTAGGCTGGAGCTGCTTC |
| 669_yobF_rv | CCTTTAATCTTTGCCATTTGAAAAATTCCTTAGATTGTTTTCTTCGCCCGGCTTGCATATGAATATCCTCCTTAG |
| 670_ptsP_fw | AAAACGCATCTGCTTATCGACGTAAAAGAGGTTAAGTCACGCCAATTATGTGTAGGCTGGAGCTGCTTC |
| 671_pstP_rv | ACCGTTAAAAGATATGTATATGATCCGCGCTATAACCCTCCGCGAATCAGGCTTGCATATGAATATCCTCCTTAG |
| 672_ackA_fw | TGGCTCCCTGACGTTTTTTTAGCCACGTATCAATTATAGGTACTTCCATGTGTAGGCTGGAGCTGCTTC |
| 673_ackA_rv | GCACCGCCAGCTGAGCTGGCGGTGTGAAATCAGGCAGTCAGGCGGCTCGCGCTTGCATATGAATATCCTCCTTAG |
| 674_yciW_fw | CCGCCTTGCGTCAGGATAACGATTTCCTTTACGACCAAGGAGCGCCCATGTGTAGGCTGGAGCTGCTTC |
| 675_yciW_rv | TTTACGCGAATCTGTCTGACGCGGCAAGGTTAATATGTCTCACCCAACGCGCTTGCATATGAATATCCTCCTTAG |
| 975_rfe_seq_fw | ACTGGCAATGACCAAGACCA |
| 976_rfe_seq_rv | CCCCAGCATATTCACCGTTG |
| 977_typA_seq_fw | ACGATGTGAATGTTGCACCA |
| 978_typA_seq_rv | GTCATACGTTTAATGCCCAGC |
| 979_yciW_seq_fw | TCGCCGTACCGTTACTATTGA |
| 980_yciW_seq_rv | TAATTCGGTCGCCATGCATG |
| 981_yobF_seq_fw | TAACTTCTCCCACTGGCCTG |
| 982_yobF_seq_rv | TCAGTGGATTCGATCAGATAGCT |
| 983_evgA_seq_fw | TGCAATACAATTCTTACGCCTGT |
| 984_evgA_seq_rv | TAAGAGGCGGCGAAGTAACT |
| 985_ptsP_seq_fw | GGTTTCTCTTGCAGCTGGTG |
| 986_ptsP_seq_rv | GCCACGGGTCCTATTGAGAA |
| 987_ackA_seq_fw | AGGTATCCTTTAGCAGCCTGA |
| 988_ackA_seq_rv | CCGTCGTGGTGGAAGAGTT |

| | |
|---|---|
| RL_rfe_colPCR_fwd | TTGCATATCAAATGGTTAATTTTTGCACAG |
| RL_rfe_colPCR_rev | CGCCAGCCCCATGCCAAT |
| RL_evgA_colPCR_fwd | CCGACTATTTATATGGTATACTTGTCG |
| RL_evgA_colPCR_rev | CGTAATTCCTTGTTGCTCAGACG |
| RL_typA_colPCR_fwd | TGACCTTTGGATAACCCTTTTTATGC |
| RL_typA_colPCR_rev | GACAAAGCTCTCTATTGACGTAACC |
| RL_yobF_colPCR_fwd2 | CTGATCGAGACATGTTTAAAAATGGC |
| RL_yobF_colPCR_rev | TTCAGCCAGAGTTTTGAAGCCATTA |
| RL_ptsP_colPCR_fwd | GTGGTGTCATTTAAACGTGATGTC |
| RL_ptsP_colPCR_rev | CTGTCACCACAAGTTCTTGTTATTTC |
| RL_ackA_colPCR_fwd | CAAAATGGCATAGACTCAAGATATTTC |
| RL_ackA_colPCR_rev | CGGTAGGGATCAGCATAATAATAC |
| RL_yciW_colPCR_fwd | CGTTCTGCCGCCCGTTATC |
| RL_yciW_colPCR_rev | TTACCACCCCTTCAGCGCG |
| 1037_evgA_phos-thio_fw | T*C*GAATTATCTTAAAGGAAGCTCAG |
| 1038_evgA_phos_rv | /5PHOS/GTTGTTACTACTGATGCCACGA |
| 1039_ackA_phos-thio_fw | T*G*CAGTGCATGATGTTAATCA |
| 1040_ackA_phos_rv | GATCAGCATAATAATACGGGACAC |
| 1041_yobF_phos_fw | GATTTGTGCGTCTGATCGAGA |
| 1042_yobF_phos-thio_rv | C*A*TCAGCCGGAGTAATGAAGC |
| 1043_ptsP_phos_fw | /5PHOS/CACTGCAGGAAAATACGCCA |
| 1044_ptsP_phos-thio_rv | G*C*AGGTTTCAGGCGCTCC |
| 1045_rfe_phos_fw | ATGGTTAATTTTTGCACAGGACT |
| 1046_rfe_phos-thio_rv | C*C*ACAAGGTACGAAACAACC |
| 1047_typA_phos_fw | ACTTGTTTGCGGAGGATGTT |
| 1048_typA_phos-thio_rv | A*C*AGACTTATAAGGGAGATACTCGCA |
| 1049_yciW_phos-thio_fw | C*C*GCCCGTTATCTCTGTTAT |
| 1050_yciW_phos_rv | CTAGCAGCGGGTTGTCCT |
| 1079_rfe50-BL21-fw | CTTCGTGGTTATACTTCTGCCA |
| 1080_rfe50-phos-thio-rv | A*G*GCATTGGTTGTGTCATCAC |
| 1081_typA50fw | TAATCCGTGTACAATAACGCGCT |
| 1082_typA50-phos-thio-rv | G*G*CAGGTTTTTTATGACTAAAAAACGA |
| 1083_evgA50-phos-thio-fw | A*T*TACTACAGGGAGAAGGGAGATGCT |
| 1084_evgA-MG-BL-50rv | GAAAAATATAGGGTAAAAACTTCATGTGG |
| 1085_evgA-W-50rv | GAAAAAAATAGGGTAAAAACTTCATGTGA |
| 1086_ackA50-phos-thio-fw | T*G*GCTCCCTGACGTTTTTTTAGC |
| 1087_ackA50rv | GCACCGCCAGCTGAGCTG |
| 1088_yciW50-phos-thio-fw | C*C*GCCTTGCGTCAGGATAAC |
| 1089_yciW50rv | TTTACGCGAATCTGTCTGACG |
| 1090_yobF50fw | TTGTATGTGATAACAGATTTCGGGT |
| 1091_yobF-phos-thio-50rv | C*C*TTTAATCTTTGCCATTTGAA |
| 1092_ptsP50fw | AAAACGCATCTGCTTATCGACG |
| 1093_ptsP-phos-thio-50rv | A*C*CGTTAAAAGATATGTATATGATCCGC |

| **Primers for Plasmids** | **Sequence** |
|---|---|
| flp_U_pkd46_rv | AACCTTATAUGCGTCTATTTATGTAGGATGAAAGG |
| rha_flp_U_fw | AGAAATACTAGAUGCCACAATTTGGTATATTATGT |
| rhamSD_AT_U_rv | ATCTAGTATTTCUCCTCAATTTCATTACG |
| RhaRS_pkd46-U_fw | ATCTGATTAAUCTTTCTGCGAATTGAGATGAC |
| pkd46_rha_U_rv | ATTAATCAGAUCCTTCCGTATTTAGCCAGT |
| pkd46_flp_U_fw | ATATAAGGTUCTTATGGCTCTTGTATCTATCAGTG |
| 795_EC_Tn7_down_U_fw | AGTCAGGUAAATGGATGCCCTGCGTAAG |
| 508_p169-rv | ACGAACGUCGGCCTCCTGTGTGAAATTG |
| 833_p8-U_fw | ACGTTCGUACTTTTCATACTCCCGCCATTCA |
| 834_p8_U_rv | AGCAAGCUACTGGCCTCCTGATGTCGTC |
| 888_p222-U-fw | ACCTGACUTATTTCAGGAAAGTTTCGGAGGAGATAG |
| 889_p222-U-rv | AGCTTGCUTCGTAATTATTGGGGACCCCTG |
| 654_pSIJ180_frt-kan-U-fw | ATGCAAGCUTTCCGTCACAGGTAGGCG |
| 655_pSIJ180_frt-kan-U-rv | ATGAAGUTCCTATTCCGAAGTTCC |
| 656_pSEVA_U_fw | ACTTCAUGGGTCCCCAATAATTACGATT |
| 657_pSEVA_gm_U_rv | ACGCGAUAGCGGAAAAGGACAACGC |
| 754_seva_spec_frt_U_rv | AGCTTGCAUAGCGGAAAAGGACAAGGGT |
| 648_pkd3-4-frt_EMG-U_fw | ATCTGAAUGTGTAGGCTGGAGCTGCTTC |
| 649_pkd3-4-frt_EMG-U_rv | AGCTTGCAUATGAATATCCTCCTTAGTTCCTATTCCGA |
| 652_pSW-amp-U_fw | AGTCCCGCUTACCAATGCTTAATCAGTGAGGCAC |
| 653_pSW-amp-U_rv | AGCACUCTGACGCCGTTGGATACACC |
| 411_pEMG_U_fw | ATGCAAGCUTCTAGGGATAACAGG |
| 651_pEMG_amp_U_rv | AGCGGGACUCTGGGGTTC |
| 650_pEMG_amp_U_fw | AGTGCUTGCGGCAGCGTG |
| 412_pEMG_U_rv | ATTCAGAUTACCCTGTTATCCCTATACTG |
| 887_pSW-508-fw | ACGTTCGUTATTTCAGGAAAGTTTCGGAGGAGATAG |
| 836_pSW1-U_rv | ATGCGCUCCTTCTTCGGCTACGTTCGTAATCA |
| 454_xylS_pm_U_fw | AGCGCAUACCTGCAGGCATGCAAGCT |
| 508_p169-rv | ACGAACGUCGGCCTCCTGTGTGAAATTG |
| 793_EC_Tn7_up_U_fw | ATCTGAAUGAGCCGTATTGAGCAGATGCT |
| 794_EC_Tn7_up_U_rv | AAACCCGUTACTCAACCGTAACCGATTTTGC |
| 795_EC_Tn7_down_U_fw | AGTCAGGUAAATGGATGCCCTGCGTAAG |
| 796_EC_Tn7_down_U_rv | AGCTTGCAUACCCTGGTAGTTAACTTTATTACCGGT |
| 411_pEMG_U_fw | ATGCAAGCUTCTAGGGATAACAGG |
| 412_pEMG_U_rv | ATTCAGAUTACCCTGTTATCCCTATACTG |
